# Strength Through Unity: The Power of the Mega-Scaffold MACF1

**DOI:** 10.3389/fcell.2021.641727

**Published:** 2021-03-18

**Authors:** Rebecca Cusseddu, Amélie Robert, Jean-François Côté

**Affiliations:** ^1^Montreal Clinical Research Institute, Montreal, QC, Canada; ^2^Molecular Biology Programs, Université de Montréal, Montreal, QC, Canada; ^3^Department of Medicine, Université de Montréal, Montreal, QC, Canada; ^4^Department of Biochemistry and Molecular Medicine, Université de Montréal, Montreal, QC, Canada; ^5^Department of Anatomy and Cell Biology, McGill University, Montreal, QC, Canada

**Keywords:** ACF7, spectraplakin, signaling, cancer, cytoskeleton

## Abstract

The tight coordination of diverse cytoskeleton elements is required to support several dynamic cellular processes involved in development and tissue homeostasis. The spectraplakin-family of proteins are composed of multiple domains that provide versatility to connect different components of the cytoskeleton, including the actin microfilaments, microtubules and intermediates filaments. Spectraplakins act as orchestrators of precise cytoskeletal dynamic events. In this review, we focus on the prototypical spectraplakin MACF1, a protein scaffold of more than 700 kDa that coordinates the crosstalk between actin microfilaments and microtubules to support cell-cell connections, cell polarity, vesicular transport, proliferation, and cell migration. We will review over two decades of research aimed at understanding the molecular, physiological and pathological roles of MACF1, with a focus on its roles in developmental and cancer. A deeper understanding of MACF1 is currently limited by technical challenges associated to the study of such a large protein and we discuss ideas to advance the field.

## Introduction

The cytoskeleton dictates the basic structure of the cell and is primarily composed of three polymers that have specific compositions and properties: the actin microfilaments (F-actin), the microtubules (MTs) and the intermediate filaments (IFs). An efficient crosstalk between the diverse components of the cytoskeleton is essential to support processes required for development and tissue homeostasis such as the establishment and maintenance of cell shape and polarity, cell-cell junctions, cell-substrate adhesion, cell migration and cell division (Fletcher and Mullins, 2010). Because these cellular processes are dynamic, they rely on the capacity of the cytoskeleton components to rapidly assemble, disassemble or translocate to specific areas of the cell and also on modulable molecular connections between them for efficient remodeling. Such a complex integrative role is fulfilled at least in part by members of the spectraplakin family, which are very large intracellular proteins (ranging from 500 to 800 kDa) composed of multiple domains that provide the capacity to bind the three major components of the cytoskeleton.

The spectraplakins combine characteristics of both the spectrin and plakin families of proteins and are evolutionarily conserved among metazoans. There are two mammalian spectraplakin genes: *Dystonin* [*DST*; also known as *Bullous pemphigoid antigen 1* (*BPAG1*) (Brown et al., 1995)] and *MACF1* [also known as *Actin cross-linking factor 7* (*ACF7*) (Byers et al., 1995; Bernier et al., 1996, 2000), *Trabeculin-*α (Sun et al., 1999), and *Macrophin* (Okuda et al., 1999)]. A single gene is present in lower organisms, including *Magellan* (Gupta et al., 2010) in *Zebrafish*, *short stop* (Lee et al., 2000) [*shot*; also known as *kakapo* (Gregory and Brown, 1998)] in *Drosophila*, and *vab-10* (Bosher et al., 2003) in *C. elegans.* Using alternative splicing and different promoters, these genes can give rise to an impressive variety of isoforms, thereby providing a powerful means to increase the functional diversity of these multifunctional proteins.

MACF1, a prototypical spectraplakin, plays a critical role in the coordination of cytoskeletal dynamics by crosslinking F-actin and MTs (Leung et al., 1999; Karakesisoglou et al., 2000). Notably, MACF1 belongs to a subset of MT plus-end tracking proteins (+ TIPs) that accumulates at the growing end of the MTs. Therefore, whether associated or not with actin filaments, MACF1 can capture MTs and sculpt the cytoskeleton (Kodama et al., 2003). MACF1 is essential for embryonic development (Leung et al., 1999; Bernier et al., 2000) where its roles in cell migration (Kodama et al., 2003; Wu et al., 2008, 2011; Margaron et al., 2013; Ka et al., 2014, 2017; Yue et al., 2016), cell proliferation (Hu et al., 2015) and signaling (Chen et al., 2006) have been demonstrated. There is also a growing body of evidence for its involvement in various pathological conditions such as genetic forms of neuromuscular diseases (Jorgensen et al., 2014; Kang et al., 2019), neurodegenerative diseases (Goryunov et al., 2010; Wang et al., 2017, 2019) and cancer (Zaoui et al., 2010).

In this review, we will describe progress over the last two decades in understanding the molecular functions as well as physiological and pathological roles of MACF1. In addition, we will highlight the challenges ahead in defining the functions of MACF1, including its biochemical properties, its regulation by phosphorylation and the ubiquitin system, and how it can serve as a signaling platform through its poorly defined interactome.

## MACF1 Isoforms and Their Structural Characteristics

**FIGURE 1:**
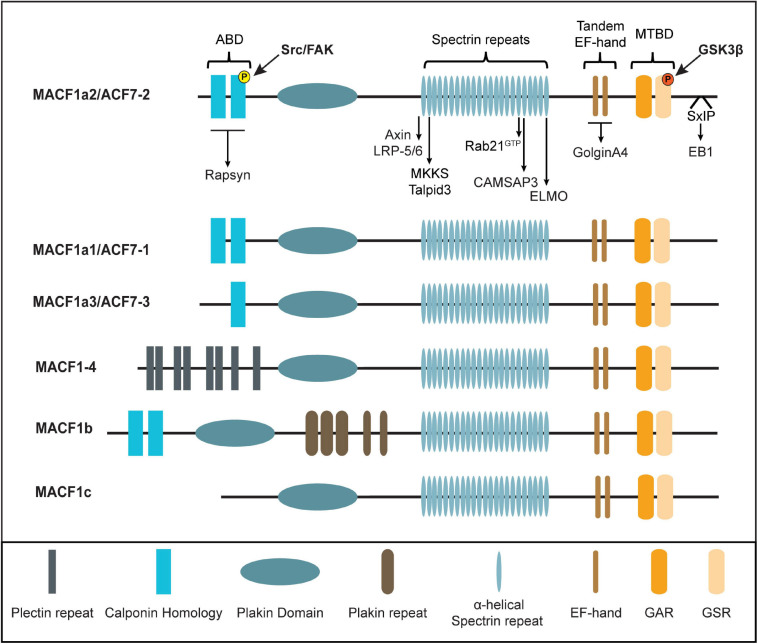
Mammalian MACF1 isoforms and interactors partners. Schematic representation of MACF1 isoforms. Direct interactors are highlighted. ABD, Actin-Binding-Domain; MTBD, Microtubules-Binding-Domain; GAR, Growth arrest-specific 2; GSR, Glycine-Serine-Arginine repeats; SxIP, Ser-x-Ile-Pro, Phospho-tyrosine in yellow and Phospho-serine in red.

The *MACF1* gene is located on mouse chromosome 4 57.42cM and human chromosome 1p34.3, and is composed of 105 and 93 exons, respectively (Bernier et al., 1996; Gong et al., 2001). Murine *Macf1* has three different transcription start sites producing several isoforms with unique structure and function (Roper et al., 2002). At least six mammalian *MACF1* isoforms with varying degrees of tissue-specific expression have been described to date (Figure 1 and Table 1). With the exception of *MACF1a2*, *MACF1-4* and *MACF1b* (Okuda et al., 1999; Sun et al., 1999; Gong et al., 2001), the tissue distribution for each of the human MACF1 isoform remains poorly documented. Each isoform is constituted of seven types of functional motifs regrouped in three main structural elements: a variable N-terminal domain, central region composed of a plakin domain and a spectrin repeat-containing rod domain and a C-terminal MT-binding domain. Unique arrangements of these motifs by RNA splicing or transcription start site variation confers to each isoform its own functional characteristics.

**TABLE 1:** Tissue distribution of mammalian MACF1 isoforms.

**Isoforms**	**Species**	**Tissue distribution**	**MW (kDa)**	**References**
MACF1-a1	*Mus musculus*	Broadly expressed (high level in skin, kidneys and stomach)	∼600	Bernier et al., 1996, 2000; Lin et al., 2005
MACF1-a2	*Mus musculus*, *Homo sapiens*	Broadly expressed (high level in brain, spinal cord and lungs)	∼600	Bernier et al., 1996, 2000; Okuda et al., 1999; Sun et al., 1999; Lin et al., 2005
MACF1-a3	*Mus musculus*	Predominant in brain and spinal cord	∼600	Bernier et al., 1996; Lin et al., 2005
MACF1-4	*Homo sapiens*	Broadly expressed (high level in heart, lungs, pituitary gland and placenta)	670	Gong et al., 2001
MACF1b	*Mus musculus*, *Homo sapiens*	Broadly expressed (high level in lungs)	∼800	Okuda et al., 1999; Sun et al., 1999; Lin et al., 2005
MACF1c	*Mus musculus*	Nervous system	∼600	Goryunov et al., 2010

The N-terminal region of the protein is the most variable among the isoforms. Most MACF1 isoforms contain two calponin homology (CH) domains, CH1 and CH2, which constitute a functional actin-binding domain (ABD) (Bernier et al., 1996; Jefferson et al., 2004). The 3-dimensional structure of the N-terminal region of murine MACF1 reveals a closed conformation of the tandem CH within the ABD (Yue et al., 2016). The phosphorylation of tyrosine 259 in the CH2 domain by the Src/FAK complex is essential for opening the conformation of MACF1, thereby allowing its interaction with F-actin. Notably, the MACF1a3 isoform has only a partial ABD (only CH2) (Bernier et al., 1996), while MACF1-4 (Gong et al., 2001) and MACF1c (Goryunov et al., 2010) have no ABD, which results in the lack of a proper F-actin-binding activity (Leung et al., 1999; Karakesisoglou et al., 2000; Jefferson et al., 2004). Therefore, the ability of MACF1 to properly crosslink F-actin and MTs is directly correlated to the presence of both the CH1 and CH2 domains with binding being regulated by phosphorylation. In MACF1-4, the ABD is replaced by 8 plectin repeats (Gong et al., 2001). Plectin motifs are generally recognized for their abilities to interact with intermediate filaments (vimentin, desmin, and keratins) (Wiche et al., 1993; Schroder et al., 1999), suggesting that MACF1-4 could function as a crosslinker between IFs and MTs despite its lack of F-actin binding. However, this potential function requires further investigation. On the other hand, the ABD of MACF1 has shown to be versatile since it can also bind to the postsynaptic scaffolding protein Rapsyn while still engaging in F-actin binding (Antolik et al., 2007; Figure 1 and Table 2).

**TABLE 2:** MACF1 direct interactors cited in this review.

**Protein**	**Description**	**Binding site**	**Methodology of identification**	**References**
Rapsyn	Post synaptic scaffolding protein	MACF1 ABD binds to the tetratricopeptide repeat domains of Rapsyn	Blot overlay and surface plasmon resonance experiments	Antolik et al., 2007
Axin	Member of the Wnt pathway complex	MACF1 Spectrin repeat 0 binds to Axin	GST-pull-down assay	Chen et al., 2006
LRP5/6	Wnt pathway co-receptor	MACF1 Spectrin repeat 0 binds to Axin	GST-pull-down assay	Chen et al., 2006
MKKS	Basal body protein	MACF1 spectrin repeat 2 binds to MKKS	Yeast two-hybrid screen using MKKS as a bait	May-Simera et al., 2009
Talpid3	Ciliary protein	MACF1 spectrin repeat 2 binds to Talpid3	Yeast two-hybrid screen using Talpid3 as a bait	May-Simera et al., 2016
Rab21_*GTP*_	Rab GTPase involved in membrane trafficking	MACF1 spectrin repeat18 binds to GTP-loaded Rab21	Yeast two-hybrid screen using Rab21 Q78L (active form) as a bait	Burgo et al., 2012
CAMSAP3	Microtubules Minus-end binding protein	MACF1 spectrin repeat 19 binds to CAMSAP3	BioID on MACF1 Spectrin repeat 19 Co-IP of CAMSAP3 with MACF1 central domain	Ning et al., 2016; Noordstra et al., 2016
ELMO	Regulator of Rac biological effects by ELMO/Dock180	MACF1 last spectrin repeat binds to C-terminal polyproline region of ELMO	Yeast two-hybrid screen using ELMO as a bait	Margaron et al., 2013
GolginA4	Trans Golgi Network protein	MACF1 fragment including Tandem EF-hand binds to the flexible N-terminal of GolginA4	Yeast two-hybrid screen using p230 as a bait	Kakinuma et al., 2004
EB1	Microtubules Plus-Tips binding protein	SxIP motif in the C-terminal domain of MACF1/MACF2	X-ray crystallography	Slep et al., 2005

Two defining features are present in all of the known isoforms of MACF1 that have led to its classification as a spectraplakin: the plakin domain and spectrin repeats contained in the central portion of the protein. Plakin domains, characterized by a high α-helical content, usually function as platforms for protein-protein interactions, especially with components of cellular junctions [reviewed in Leung et al. (2002)]. Such a function was demonstrated for the plakin domain of the related Dst (BPAG1), conferring its ability to bind hemidesmosomes (Koster et al., 2003). Although MACF1 was observed to be present at the tight junctions of intestinal epithelium (Ma et al., 2017), the involvement of its plakin domain for this recruitment has yet to be demonstrated. Furthermore, a weak MT-binding affinity has been attributed to the plakin domain of MACF1 (Karakesisoglou et al., 2000), but the biological relevance of this activity will require further investigation. The other feature of the central portion of MACF1 is composed of dystrophin-like spectrin repeats, each containing between 110 and 120 residues that form a three-helix bundle (Yan et al., 1993; Leung et al., 1999). Crystallographic analyses revealed that the spectrin repeats of the protein spectrin form an extended rod-like structure that constitutes a flexible and elastic linker between the actin and MT binding functional domains found in the N-terminus and C-terminus, respectively (Yan et al., 1993; Pascual et al., 1997). The spectrin repeats are responsible for the interaction with a plethora of signaling proteins, including Wnt pathway components, ciliary proteins, CAMSAP3, ELMO and the GTP loaded form of Rab21, thereby conferring a signaling platform function to this large protein (Chen et al., 2006; May-Simera et al., 2009, 2016; Burgo et al., 2012; Margaron et al., 2013; Ning et al., 2016; Noordstra et al., 2016; Figure 1 and Table 2). MACF1b, one of the longest transcript, encodes an extra plakin repeat domain (PRD) between the canonical plakin domain and the spectrin repeats (Lin et al., 2005). The PRD is predicted to confer the ability to bind intermediate filaments, as seen for BPAG1 (Fontao et al., 2003) and desmoplakin (Lapouge et al., 2006), but this remains to be shown for MACF1. Intriguingly, this extra PRD has been shown to target MACF1b to the Golgi (Lin et al., 2005); but the biological relevance of this specific localization remains to be fully defined.

The C-terminal portion of the protein is common to all of the known isoforms of MACF1 and it is composed of: (1) two EF-hand motifs (EF1-EF2), (2) a growth arrest-specific 2 (GAR) domain that is strictly restricted to the spectraplakin family (Roper et al., 2002), and (3) a domain containing Glycine-Serine-Arginine repeats (GSR) (Leung et al., 1999; Sun et al., 2001). The role of the EF-hand motifs in MT-binding has been controversial. Although one study suggests a crucial role in mediating MT-binding (Kapur et al., 2012), other investigations have attributed the MT-binding largely to the GAR and GSR module (Sun et al., 2001). Structural analyses of the EF1-EF2-GARh fragment of MACF1 revealed that the EF1-EF2 and GAR domains are connected by a flexible linker (Lane et al., 2017). Functional analyses of the EF1-EF2-GAR module demonstrated that the EF-hand motif was not sufficient to bind MT, while the GAR domain is. The presence of the GAR domain enhanced the ability of the EF1-EF2 domain to bind the MT lattice, confirming the predominant role of the GAR domain in MT-binding (Lane et al., 2017). Interaction of the C-terminus of MACF1 with a number of other proteins has also been documented (Figure 1 and Table 2). For example, the trans-Golgi resident protein GolginA4 binds directly to a region overlapping with the two EF-hands of MACF1, without interfering with the MT-binding activity of the protein (Kakinuma et al., 2004). Finally, the C-terminus part of MACF1 harbors a Ser-x-Ile-Pro (SxIP) motif, a signature motif for binding of MT plus-end-tracking proteins (+ TIPs) such as EB1 (Slep et al., 2005). It will be important to determine how these interactions contribute to the MT plus-end tracking activity of MACF1 and how they are regulated.

## Signaling by MACF1 in Physiological Conditions

Through its capacity to crosslink cytoskeletal elements and recruit a variety signaling molecules, MACF1 plays a key role in cellular functions involved in embryonic development and tissue homeostasis in adults.

### MACF1 in Embryonic Development

**FIGURE 2:**
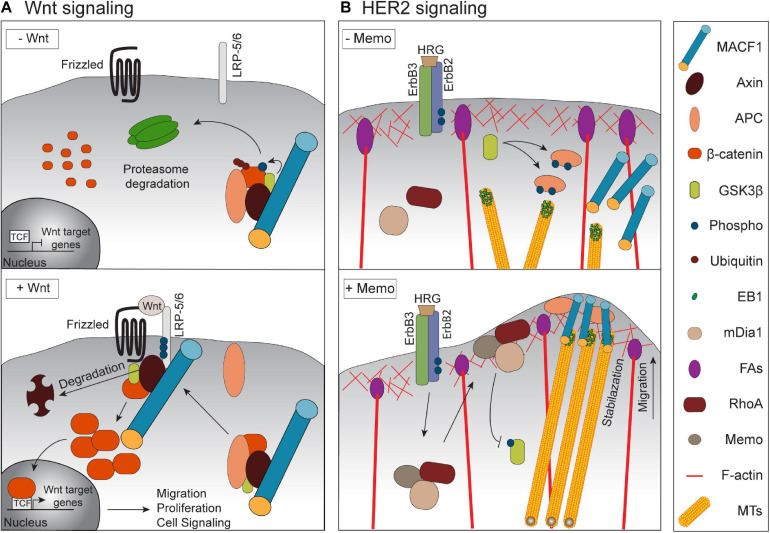
Signaling by MACF1 in physiological and cancer context. **(A)** Upon Wnt stimulation, MACF1 is required for a proper translocation of the Axin/APC/GSK3β/β-catenin complex to the membrane. The subsequent degradation of Axin permits the translocation of β-catenin into the nucleus and the induction of migration, and proliferation -related genes. **(B)** Upon activation of the HER2 receptor by its ligand HRG, the complex Memo-RhoA-mDia localizes at the leading edge and inhibits GSK3β activity. This inhibition prevents APC phosphorylation and allows the recruitment of MACF1 that stabilizes MTs at the cell cortex.

*MACF1* is broadly expressed in embryonic tissues and is detectable as early as embryonic day 7.5 during murine development (Bernier et al., 2000). Notably, isoform distribution is tissue-specific, and the levels of expression of these isoforms depend on the embryonic developmental stage. MACF1 expression is predominant in the nervous system and skeletal muscle during development with the highest levels in the headfold and the primitive streak (Chen et al., 2006). Two independent laboratories have generated global *MACF1*-null mouse models in order to study its role in embryogenesis. In both models, the deletion of *MACF1*, which was predicted to affect all of its isoforms, led to early embryonic lethality (Kodama et al., 2003; Chen et al., 2006). Notably, MACF1-null embryos failed to form the primitive streak and node, two structures involved in gastrulation, and this resulted in a specific absence of the mesoderm lineage since ectoderm and endoderm derivatives were present (Chen et al., 2006). The authors found striking phenotypic similarities between *MACF1*-null animals and knockout mice for components of Wnt signaling, namely the Wnt3 ligand and LRP5/6 that are Wnt co-receptors together with Frizzled receptors. In cell culture conditions, Wnt1 stimulation promoted the formation of a MACF1 multiprotein complex at membranes that included the Wnt pathway components Axin, β-catenin, GSK3β and LRP6, mediated by the direct binding of MACF1 to Axin and the co-receptor LRP5-6 (Figure 2A). Depletion of MACF1 by siRNA dampened Wnt1-induced β-catenin transcriptional activity, therefore establishing a connection between MACF1 and Wnt signaling (Chen et al., 2006). The authors found that the Wnt-MACF1 signaling axis was important to ensure the production of the *Brachyury* transcript, a key developmental gene involved in both the formation of the primitive streak and node as well as for gastrulation, therefore providing a potential explanation for the observed phenotypes in *MACF1*-null embryos. A contribution of MACF1 in other developmental Wnt signaling contexts has surprisingly not been reported; however, such studies will require the generation of tissue specific *MACF1* knockout models. Notably, although a recent genome-wide cellular CRISPR screen performed in pancreatic ductal adenocarcinoma cells that rely on Wnt signaling for proliferation (Steinhart et al., 2017) identified a number of core genes required for the propagation of Wnt signals, *MACF1* was not among them. Whether MACF1 is a general contributor to Wnt signaling or if it instead acts in specialized cellular systems (such as embryonic and stem cells) or context-specific diseases remains to be determined.

**FIGURE 3:**
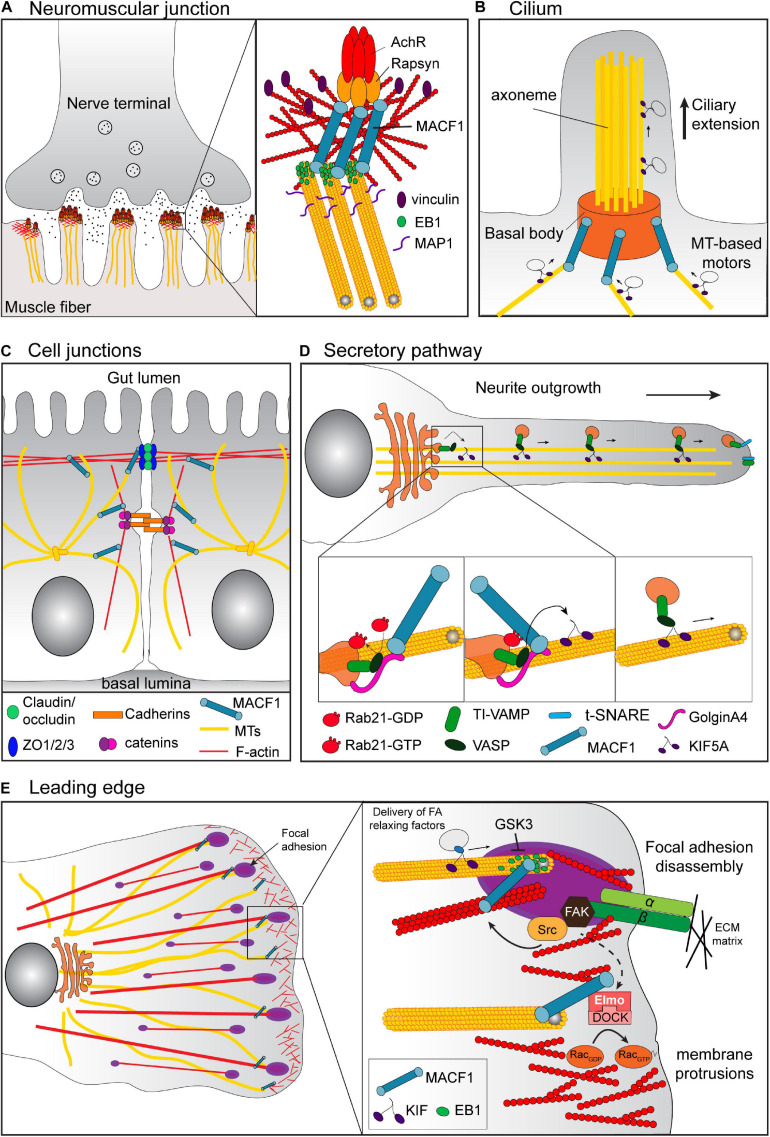
Cell context-specific functions of MACF1. **(A)** At the neuromuscular junction, MACF1 promotes the stabilization of the AChR at the actin-rich postsynaptic membrane via its binding to Rapsyn and the recruitment of the EB1/MAP1b/Vinculin/βtubulin complex. **(B)** In ciliated cells, MACF1a connects the MTs with the basal body of the cilia via its interaction with Talpid3 and MKKs (not represented). This MT anchoring at the basal body is required for the docking of ciliary vesicles involved in cilium elongation. **(C)** In the intestinal epithelium, MACF1 participates in the maintenance of the intestinal barrier. MACF1 connects the MTs to ZO-1 positive tight junctions, affecting their dynamics by an unknown mechanism. **(D)** MACF1 is also involved in vesicular trafficking from the TGN through the plasma membrane via its interaction with GolginA4. For example, in hippocampal neurons, the complex of GTP-loaded Rab21, MACF1 and GolginA4 is required for the KIF5A-dependent transport along MT of TI-VAMP v-SNARE-tagged vesicles from the Golgi to neurite tips, which is essential for the axonal growth. **(E)** At the leading edge of migratory cells, MACF1 connects the growing MT tips to the FA to regulate FA turnover. MACF1 also induces the recruitment of the complex Elmo/DOCK that activates RAC1 to promote actin polymerization required for membrane protrusion.

The use of tissue-specific knockout mice has highlighted the roles of MACF1 in several tissues. Nervous system-specific *MACF1* knockout mice died within 24–36 h after birth and displayed several defects in brain structures that were associated with impaired neurite outgrowth and neuronal migration (Goryunov et al., 2010; Ka et al., 2014). Skeletal muscle-specific *MACF1* knockout mice displayed impaired neuromuscular synapses and aberrant sarcomeric reticulum and mitochondrial structures that were associated with a decrease in muscle activity and a reduction in muscle fiber size after birth, even though the early development of the neuromuscular junction occurred normally (Oury et al., 2019). MACF1 is linked to the acetylcholine receptor (AChR) via its binding to Rapsyn (Antolik et al., 2007). In the absence of MACF1 at the actin-rich postsynaptic membrane, the density of the AChRs at the neuromuscular junction decreases gradually as it is no longer stabilized by the EB1/MAP1b/Vinculin/β-tubulin complex normally recruited by MACF1 (Oury et al., 2019; Figure 3A). Retina progenitor-specific MACF1 knockout mice revealed a crucial role for MACF1a in ciliogenesis, which appeared to be conserved in other ciliated cells (May-Simera et al., 2016; Figure 3B). MACF1a interacts with the ciliary proteins MKKS and Talpid3 at the basal body (May-Simera et al., 2009, 2016) and its loss impaired the cytoskeletal interactions at the basal body of the cilium. For instance, the lack of MT anchoring at the basal body caused by the loss of MACF1 prevents the docking of ciliary vesicles required for cilium elongation. Finally, conditional knockout of MACF1 in primary osteoblasts induced delayed ossification and a decrease in bone mass associated with an inhibition of Bmp2/Smad/Runx2 signaling (Qiu et al., 2020), confirming a role for MACF1 in bone development as suggested previously *in vitro* (Hu et al., 2018; Yin et al., 2018; Zhang et al., 2018).

### MACF1 in Cellular Processes

Through their ability to connect different cellular structures in a tissue-specific manner, the MACF1 isoforms exert their functions by regulating fundamental cellular processes. Therefore, MACF1 has an impact not only on embryogenesis, but also on the maintenance of tissue integrity, such as tight junction dynamics, or the establishment of cell polarity associated with proliferation, vesicular transport and cell migration.

Regulation of the cell-cell junction in the intestinal epithelium is essential for the maintenance of the intestinal barrier and a role for MACF1 in that process was established using intestinal conditional knockout mice (Ma et al., 2017; Figure 3C). Although the terminal differentiation of the gut was not altered by intestinal *MACF1* ablation, these mice showed an altered intestinal physiology with an increase of the size of villi and the depth of crypts and a less organized and polarized intestinal epithelium. At the cellular level, the absence of MACF1 decreased the tight junction dynamics associated with wound healing defects in intestinal epithelium, which increased the susceptibility of the conditional knockout mice to experimental colitis. The defects in tight junction dynamics were attributed to the dissociation of the MT network from the ZO-1-positive tight junctions in absence of MACF1. In MACF1 knockout Caco-2 cells, the rescue of tight junction dynamics and wound healing required the GAR domain of MACF1, suggesting that MACF1 regulates these processes through its interaction with MTs. However, the molecular connection between MACF1 and the tight junctions, as well as the MT-dependent mechanism responsible for tight junction dynamics and how these processes are related to colitis remains unclear.

Cell proliferation involves cooperation of MT and F-actin to regulate the positioning of the spindle and to orchestrate cytokinesis, thereby ensuring a complete cell division. In osteoblastic cells, the presence of MACF1 as a crosslinker between F-actin and MTs was found to be important for cell proliferation (Hu et al., 2015). The knockdown of MACF1 in these cells induced the disorganization of the cytoskeleton and the disappearance of the MTOC as well as the presence of binuclear/multinuclear structure associated with a S phase cell cycle arrest. In contrast, in epidermis the suppression of MACF1 had no impact on cell proliferation (Kodama et al., 2003) indicating that this function could be cell-type specific. However, as MACF1 regulates centrosome movement in neurons (Ka et al., 2014) and because the correct positioning of the centrosome is necessary for dividing neural progenitors (Higginbotham and Gleeson, 2007), a role for MACF1 in neural cell proliferation has been suggested and will warrant further investigations (Moffat et al., 2017).

Consistent with its partial localization at the Golgi apparatus (Lin et al., 2005), MACF1 was also shown to be involved in vesicular trafficking from the trans-Golgi network through the plasma membrane (Burgo et al., 2012; Sohda et al., 2015) via its interaction with the protein GolginA4 (Kakinuma et al., 2004). In hippocampal neurons, MACF1 was shown to be an effector of the small GTPase Rab21. In that context, the complex of GTP-loaded Rab21, MACF1 and GolginA4 was required for the KIF5A-dependent transport along MT of TI-VAMP v-SNARE-tagged vesicles from the Golgi to neurite tips, which is essential for axonal growth (Burgo et al., 2012; Figure 3D). The interaction of MACF1 with GolginA4 also played a crucial role during autophagy (Sohda et al., 2015) and is necessary for the trafficking of the autophagy-related protein mATG9 from the trans-Golgi to the cell surface. As a consequence, MACF1 knockdown impairs phagophore formation, an early step in autophagy in the context of amino acid starvation.

The best documented function of MACF1 remains its ability to facilitate cell migration. To sustain cell migration, cells acquire a polarized morphology and develop a single leading edge by reorganizing the F-actin and MTs that provide the mechanical strength necessary for this process. Given its crucial role in the coordination of cytoskeletal dynamics (Kodama et al., 2003), it is not surprising that MACF1 has been shown to play a role in cell migration in several contexts (Kodama et al., 2003; Wu et al., 2008, 2011; Margaron et al., 2013; Ka et al., 2014, 2017; Yue et al., 2016; Figure 3E). This function was first discovered by inactivating *MACF1* in the epidermis where its loss causes defective polarization of stable MTs and aberrant cell migration after wounding (Kodama et al., 2003). MACF1 links the growing ends of MTs and was found to be enriched at the leading edge of polarized epidermal cells. Loss of MACF1 altered the MT dynamics and led to a disorganization of the MT network. The MTs were less stable and exhibited a random orientation, inducing a dissociation with the actin bundles that resulted in abnormal cell migration during wounding of the tissue. In skin stem cells, the regulation of MT-MACF1 interaction via GSK3β was found to affect directed migration. The phosphorylation of residues in the GSR domain of MACF1 by GSK3β uncoupled MACF1 from MTs and altered MT directionality, cell polarity and migration (Wu et al., 2011). The rapid inhibition of GSK3β activity by upstream signaling, such as Wnt activators or HER2 (ErbB2) activation, led to localized dephosphorylation of the MACF1 C-terminus to allow a proper MT polarity necessary to promote cell migration (Chen et al., 2006; Zaoui et al., 2010). A connection between MACF1 and GSK3 signaling is also found in neuronal migration in the developing cortex. In nervous-system-specific MACF1 knockout mice, the deletion of MACF1 in developing neurons induces aberrant dynamics at the leading edge and the centrosome, leading to defect in pyramidal neuron migration (Ka et al., 2014). MACF1 phosphorylation by GSK3 dissociated MACF1 from MT and inhibited its role in neuronal migration (Ka et al., 2014). Whether additional kinases are involved in regulating the MT-binding activity remains to be explored, and conversely, the identity of phosphatases ensuring localized dephosphorylation of the GAR domain have remained elusive.

In addition to GSK3, MACF1 is also a substrate for the FAK/Src tyrosine kinases complex at focal adhesions (Yue et al., 2016). MACF1-deficient endodermal cells fail to properly target MTs to focal adhesions (FAs) resulting in the stabilization of those complexes (Wu et al., 2008). It has been suggested that MACF1 guides the growing MTs along the actin filaments toward FAs, which allows the MT motor molecules to deliver relaxing factors that promote the regulation of FA turnover (Wu et al., 2008). FAK/Src-dependent phosphorylation of MACF1 is required for its binding to F-actin, its coordination of actin dynamics at focal adhesions and its pro-migratory role in skin epidermis (Yue et al., 2016). MACF1 is also robustly expressed in osteoblasts and can regulate preosteoblast migration by mediating FA turnover through the + TIPs EB1 (Su et al., 2020). MACF1 enhances preosteoblast polarization and migration by increasing MT stabilization at FAs. It has been suggested that MACF1 diminishes EB1 interaction with APC at focal adhesions by increasing Src activation and Src-dependent EB1 phosphorylation.

Another mechanism by which MACF1 can promote and sustain cell migration is through its interaction with the human engulfment and cell motility protein ELMO1 (Margaron et al., 2013). ELMO recruits MACF1 at the membrane and promotes MT capture and stabilization at the leading-edge, thereby inducing formation of membrane protrusions. The ELMO/MACF1 complex coordinates MT dynamics and actin polymerization at the leading edge through its recruitment of DOCK180 and by a spatiotemporal activation of Rac near MT-growing ends (Figure 3E).

## Signaling by MACF1 in Cancer and Other Diseases

### Genetic Alterations of MACF1 Associated With Diseases

The first reported mutation of the *MACF1* gene was discovered in 2014 in a family affected by neuromuscular conditions including periodic hypotonia, lax muscles, diminished motor skills, and a joint contracture (Jorgensen et al., 2014). The genetic defect arose from a heterozygous duplication that caused lower expression of the mRNA and protein product. More recently, two novel heterozygous missense mutations caused by the amino acid substitutions Thr506Ile and Ile3885Thr, were reported in a Chinese family afflicted by similar neuromuscular conditions (Kang et al., 2019). However, this novel type of myopathy, termed “spectraplakinopathy,” may only be the tip of the iceberg of conditions caused by MACF1 mutations. Consistent with the expression of brain specific MACF1 isoforms, there is a growing list of MACF1 genetic alterations that have been associated with susceptibility to diverse neuropathologies and particularly psychological disorders such as schizophrenia (Kenny et al., 2014; Wang et al., 2015), autism spectrum disorder (Kenny et al., 2014), and bipolar disorders (Han et al., 2019) as well as neurodegenerative diseases such as Parkinson’s (Wang et al., 2017). Heterozygous missense mutations within the MACF1 GAR domain coding region also cause defects in neuronal migration and axon guidance, which result in a rare form of brain malformation called lissencephaly (Dobyns et al., 2018). The current genomics efforts to identify mutated genes in rare diseases will likely reveal new roles for MACF1 in the future.

### MACF1 and Cancer

In addition to the growing list of pathologies associated with *MACF1* genetic alterations, aberrant expression of *MACF1* is also related to various human cancers. Because of its role in cell migration, invasion and proliferation, investigations are ongoing to evaluate its role in tumor growth and metastasis and to evaluate whether it could be a candidate for targeted therapeutics.

Due to its high expression in the brain and its function as a cytoskeletal cross-linking protein, the role of MACF1 was investigated in brain tumors such as glioblastoma (GBM). Careful analysis of GBM from different stages revealed that MACF1 is highly expressed in grade IV GBM but not in surrounding normal tissues or lower grade GBM, revealing its potential as a prognostic marker for this type of cancer (Afghani et al., 2017). To further investigate its function in this context, downregulation of MACF1 was performed with RNA interference in both glioblastoma cell lines and patient derived xenograft cell lines. As expected, loss of MACF1 inhibited cell migration, but it also inhibited cell proliferation by blocking the cell cycle progression. Consistent with the role of MACF1 in Wnt signaling (Chen et al., 2006), these anti-tumorigenic effects were also associated with a significant down-regulation of Wnt signaling and its mediators associated proteins, Axin1 and β-catenin, whose dysregulation is well known to be involved in tumor progression. Although chemotherapeutic agents and radiotherapy are commonly used to treat GBM, they have minimal effectiveness due to acquired tumor resistance (Ramirez et al., 2013; Khosla, 2016). In an effort to enhance therapeutic benefit, combination of traditional therapies with inhibition of MACF1 was tested in this type of cancer. The combination of two treatments with, for example, the alkylating agent temozolomide coupled with a MACF1 siRNA or combining radiation therapy with downregulation of MACF1, has been shown to significantly decrease cell viability (Afghani et al., 2017; Bonner et al., 2020). In fact, the use of temozolomide alone increases MACF1 protein level and therefore Wnt-cytoplasmic complex in cells, supporting the idea that the interaction between MACF1 and Wnt signaling contributes to resistance to temozolomide (Afghani et al., 2017). Mechanistically, the combination of radiation and MACF1 depletion results in a decreased expression of a downstream effector of the mTOR signaling pathway, the ribosomal protein S6. In addition, both MACF1 and ribosomal protein S6 had a positive correlative expression with DNA damage repair genes such as ATM or BRCA2 (Bonner et al., 2020), suggesting that the radio-sensitization effects of MACF1 inhibition are the consequence of a decrease in DNA damage repair response. Although these investigations have suggested a potential role for MACF1 in combinatorial targeted therapies to enhance progression-free survival of patients with GBM, additional *in vivo* investigations must be performed to further support these data.

The role of MACF1 as a potential metastatic regulator was also investigated both in breast cancer and melanoma (Duhamel et al., 2018; Wang et al., 2020). In the HER2 positive subtype of breast cancer it was reported that the capture of MTs at the leading edge of migrating cells was enhanced by the activation of the receptor HER2 by its ligand heregulin (HRG) via the formation of a complex that also includes Memo, RhoA and mDia1 (Zaoui et al., 2008, 2010). Both APC and CLASP2, two plus-end binding proteins (+TIPs) involved in MT stabilization, act downstream of Memo to capture MTs at the cortex via the recruitment of MACF1 (Zaoui et al., 2010). This binding was shown to be regulated by the GSK3β-mediated phosphorylation of APC and CLASP2 which prevent MT stabilization (Zumbrunn et al., 2001; Wittmann and Waterman-Storer, 2005). The results of this study suggest that HER2 activates the Memo-RhoA-mDia1 axis and inhibits the GSK3β activity, preventing the APC phosphorylation. This allows its localization at the cell cortex and the recruitment of MACF1, which is required for MT capture and stabilization (Figure 2B). Although this study highlighted for the first time a potential role for MACF1 in breast cancer (Zaoui et al., 2010), investigations in *in vivo* models are warranted to determine if MACF1 is an important contributor of HER2-induced breast cancer progression and metastasis.

Cancer cells exploit the epithelial-to-mesenchymal transition (EMT) program to gain migratory and invasive properties and become metastatic. This crucial transition is tightly controlled, notably by cytoskeletal regulators, and a dysregulation can lead to an increase in metastasis and poor prognosis for patients (Sun et al., 2015). Downregulation of MACF1 in B16F10 melanoma cells led to a decrease in cell proliferation which was confirmed *in vivo* with a decrease in tumor size compared to control cells. In addition, MACF1 knockdown induced a switch of expression in mesenchymal and epithelial biomarkers. The level of expression of N-cadherin and TGFβ was significantly decreased whereas E-cadherin and SMAD-7 were increased, suggesting a role for MACF1 in the EMT in this specific context. The knockdown of MACF1 expression in mice led to a decrease in tumorigenicity associated with decreased EMT biomarkers and fewer metastatic nodules in the recipient mouse lungs (Wang et al., 2020). These results are consistent with the capacity of MACF1 to enhance cell migration through increased invasion and metastasis.

**FIGURE 4:**
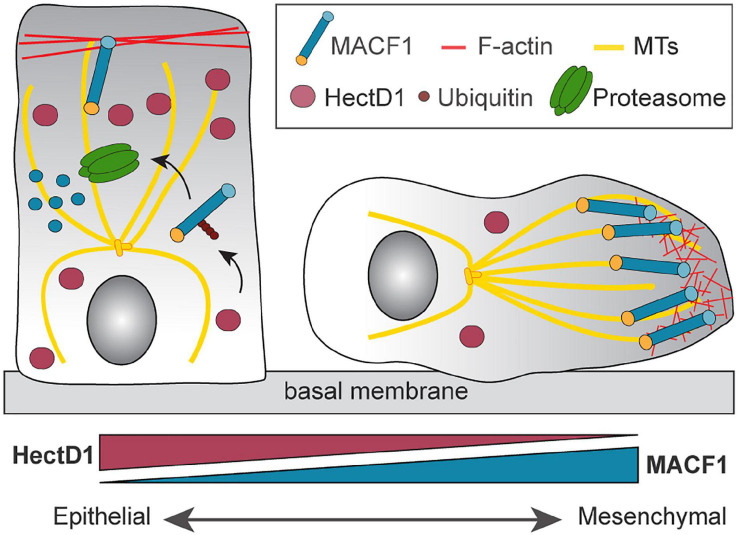
Regulation of MACF1 by the E3 ubiquitin ligase HectD1. The control of MACF1 by HectD1 controls the transition between an epithelial and mesenchymal state. E3 ubiquitin ligase HectD1 mediates the formation of Lys-48-linked ubiquitin chains on MACF1 and targets it for proteasome-dependent degradation. Down regulation of HectD1 induces the stabilization of MACF1 and contributes to the acquisition of the mesenchymal phenotype.

A systematic investigation of the roles of + TIP proteins in EMT and migration also revealed a determinant role for MACF1 in breast cancer progression (Duhamel et al., 2018). Using a shRNA screen that depleted most + TIPs in the metastatic breast cancer cell line MDA-MB-231, MACF1 was the only candidate found to play a role in both cell migration and the EMT state. In fact, MACF1 deletion was found to decrease cell migration and regulate the mRNA levels of the canonical EMT makers, thereby inducing a switch to a more epithelial state. These results were also extended to additional breast cancer cell lines representing other molecular subtypes such as Luminal A and B as well as HER2+. *In vivo* experimental metastasis assays in mice have also shown a robust decrease in lung metastasis when MACF1 was depleted, revealing a role for MACF1 in the metastatic progression of breast cancer. Affinity purification coupled to mass spectrometry was used to understand how the protein levels of MACF1 could be regulated, and these experiments revealed a novel interactor of MACF1, the E3 ubiquitin ligase HectD1. Depletion of HectD1 was found to robustly increase MACF1 protein levels, which correlated with an increase in MACF1-dependent cell migration. Conversely, overexpression of HectD1 mediated the formation of Lys-48-linked ubiquitin chains on MACF1, thus targeting it for proteasome-dependent degradation. By stabilizing MACF1 protein levels, HectD1 depletion induced an increase of mesenchymal marker expression and led to an increase in directional cell migration. Notably, depletion of HectD1 in the poorly metastatic T47D cells enhanced the number of metastatic lung lesions in comparison to control cells (Duhamel et al., 2018) suggesting a role of HectD1 in metastasis progression *in vivo*. This study highlighted the novel role of HectD1 as a regulator of MACF1 stability and raised the potential of therapeutic approaches that exploit MACF1 ubiquitination and proteasome-mediated degradation to limit metastasis (Figure 4).

## Challenges and Perspectives

Despite the impressive amount of current information available concerning the biochemistry and biological importance of MACF1, significant gaps still exist in our understanding of its regulation and function. There are at least three fundamental aspects that warrant significant experimental efforts in years to come.

### Expression of the MACF1 Isoforms and Their Functional Impacts

There is a clear need to more thoroughly catalog the expression patterns of the various isoforms at the mRNA and at the protein levels. As discussed in this review, the varying ability of the isoforms to connect the different cytoskeleton components endorse them with different functional properties. In that respect, a major question that remains is whether MACF1 could crosslink intermediate filaments and MTs and to determine the functional impact of this on cell migration and other biological processes. This task is complicated by the enormous size of the gene and the corresponding protein isoforms. For example, the difficulty of overexpressing such a big cDNA has forced investigators to overexpress truncated forms of the protein, which often includes only the N-terminal ABD and the C-terminal MTB (Kodama et al., 2003; Wu et al., 2008, 2011; Zaoui et al., 2010). Although this strategy has revealed the importance of MACF1 in coordinating the F-actin and the MT networks in different contexts, it probably doesn’t reflect the full potential of MACF1 to act as a signaling scaffold. For example, the binding of MACF1 to IFs has been often proposed (Byers et al., 1995; Jefferson et al., 2004; Lin et al., 2005) but never been investigated properly. Because of the variation in the presence of different putative IF-binding domains in murine MACF1 isoforms, it is very likely that the IF affinity of each MACF1 isoform will be different. The fact that the composition of IF is developmental- and tissue-specific (reviewed in Chung et al., 2013) raises the possibility that each MACF1 isoform could bind to a different IF type depending on the cell context. Indeed, tissue-specific MACF1 isoforms probably have distinct interactomes and the fact that most studies are based on the same cDNA used in different contexts may not accurately reflect all of their cell-specific functions. In that respect, developing a panel of antibodies that would allow the study of the cellular location and tissue distribution of the individual MACF1 isoforms would constitute a major advance. Recent technological breakthroughs may now allow the testing of the biological functions of individual isoforms of MACF1. Conditional knockout mouse models generated by the Fuchs laboratory was designed to eliminate all of the known isoforms through Cre-mediated gene targeting tissues of interest. Rescue approaches in this mouse model could provide an method to re-express individual isoforms and thereby probe their functions. Indeed, the Kothary laboratory re-expressed a single isoform of the related protein Dystonin (Dystonin-a2) in a mouse disease model lacking expression of Dystonin-a1 and Dystonin-a2, which led to partial rescue of the phenotype (Ferrier et al., 2014). Such studies are, however, quite challenging to perform and also depend on single isoform rescue. One approach for the future might be to exploit RNA editing approaches that specifically degrade the mRNAs coding for the individual isoforms of MACF1. By using the Cas13 technology (Cox et al., 2017), guide RNAs specific for all the MACF1 transcripts could be designed to target the various mRNAs alone or in combination for degradation, resulting in specific depletion of the associated protein products. Such elegant loss-of-function assays are needed to reveal the specific roles of the MACF1 isoforms in cell lines and in mouse models.

### What Is the Landscape of the Molecular Functions of MACF1 and Its Associated Proteins?

Although much is now understood about how MACF1 binds, and/or crosslinks, actin filaments and microtubules, the identity of any additional associated proteins of this mega-scaffold and the connections they bring to different cellular machineries remains an area of needed investigation. Only a handful of proteomic studies have been attempted on MACF1 (Duhamel et al., 2018). Given the large size of the protein and the lack of antibodies to specific isoforms, such biochemical analyses have been challenging. New proximity labeling approaches, such as BioID and APEX, applied to MACF1 isoforms, and in various cellular contexts, might resolve some of these biochemical challenges and reveal unprecedented insight into global interactomes of the various isoforms. Such studies will allow a better positioning of MACF1 in the larger context of the 3 major cytoskeleton components and its action during cytoskeletal dynamics.

### Structural Insights of the Various MACF1 Isoforms: From Regulation to Functions

The extremely large size of the MACF1 isoforms has precluded attempts at structural studies on the full-length proteins. Nevertheless, important recent progress has been made in solving the structure of two of the functional domains of MACF1; the actin-binding domain (ABD) and the MT-binding domain (Yue et al., 2016; Lane et al., 2017). The future challenge will be to solve the structural features of the full-length isoforms to reveal how these proteins are organized in space. Given their size, the MACF1 isoforms would be ideally suited for Cryo-EM structural analyses. Such studies could be performed on the apoprotein to reveal, for example, if the protein exists in an auto-regulated conformation through intramolecular contacts. Alternatively, such structural studies might also reveal the structure of MACF1 bound to components of the cytoskeleton. Finally, given the large number of phosphorylation sites identified in MACF1 (as per PhosphoSitePlus), structural information may be useful in determining the potential impact of these modifications.

## Author Contributions

RC, AR, and J-FC wrote the manuscript. RC and AR designed the figures. All authors contributed to the article and approved the submitted version.

## Conflict of Interest

The authors declare that the research was conducted in the absence of any commercial or financial relationships that could be construed as a potential conflict of interest.
